# Parameter Estimation in Epidemiology: from Simple to Complex Dynamics

**DOI:** 10.1063/1.3637843

**Published:** 2011-09-14

**Authors:** Maíra Aguiar, Sebastién Ballesteros, João Pedro Boto, Bob W. Kooi, Luís Mateus, Nico Stollenwerk

**Affiliations:** a Centro de Matemática e Aplicações Fundamentais CMAF, Universidade de Lisboa, Avenida Prof. Gama Pinto 2, 1649‐003 Lisboa, Portugal; b Faculty of Earth and Life Sciences, Department of Theoretical Biology, Vrije Universiteit Amsterdam, De Boelelaan 1087, NL 1081 HV Amsterdam, The Netherlands; University of Peloponnese; University of Peloponnese; Technological Educational Institute of Chalkis; School of Pedagogical & Technological Education (ASPETE)

## Abstract

We revisit the parameter estimation framework for population biological dynamical systems, and apply it to calibrate various models in epidemiology with empirical time series, namely influenza and dengue fever. When it comes to more complex models like multi‐strain dynamics to describe the virus‐host interaction in dengue fever, even most recently developed parameter estimation techniques, like maximum likelihood iterated filtering, come to their computational limits. However, the first results of parameter estimation with data on dengue fever from Thailand indicate a subtle interplay between stochasticity and deterministic skeleton. The deterministic system on its own already displays complex dynamics up to deterministic chaos and coexistence of multiple attractors.

## INTRODUCTION

A major contribution to stochasticicty in empirical epidemiological data is population noise which is modelled by time continuous Markov processes or master equations [1, 2, 3]. In some cases the master equation can be analytically solved and from the solution a likelihood function be given [4]. The likelihood function gives best estimates via maximisation or can be used in the Bayesian framework to calculate the posterior distribution of parameters [3]. Here we start with an example of a linear infection model which can be solved analytically in all aspects and then generalize to more complex epidemiological models which are relevant for the description of e.g. influenza or dengue fever [5], on the cost of having to perform more and more steps by simulation to obtain the likelihood function by complete enumeration [6] or even just to search for the maximum in extreme cases.

Recent applications to a multi-strain model applied to empirical data sets of dengue fever in Thailand, where the model displays such complex dynamics as deterministic chaos in wide parameter regions [5, 7], stretch the presently available methods of parameter estimation well to its limits. Finally, the analysis of scaling of solely population noise indicates that very large world regions have to be considered in data analysis in order to be able to compare the fluctuations of the stochastic system with the much easier to analyze deterministic skeleton, which however can already show deterministic chaos [8, 9], here via torus bifurcations [10].

## AN ANALYTICALLY SOLVABLE CASE

The simplest epidemiological model, the SI system, where infection is only aquired from the outside, leads to a master equation which is not only linear in probability but also in the state variables, leading to a linear mean field approximation for the dynamics of the expectation values [3]. Though very simple in its set-up, it can be applied to real world data of influenza in certain stages of the underlying SIR model, when considering the cumulative number of infected cases during the outbreak [4],

$$S+I^* \quad \xrightarrow{\beta} \quad I+I^*$$

*Numerical Analysis and Applied Mathematics ICNAAM 2011*
AIP Conf. Proc. 1389, 1248-1251 (2011); doi: 10.1063/1.3637843
© 2011 American Institute of Physics 978-0-7354-0956-9/$30.00

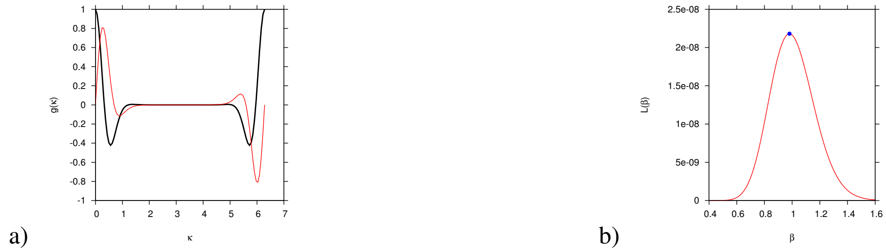

**FIGURE 1.** a) An example of the characteristic function with fixed $\Delta t$ given by the sampling rate of the time series. b) From the characteristic function we can obtain the solution of the stochastic model and with it the likelihood function, as shown here with its best estimate of the model parameter as maximum.

for infected $I$ and susceptibles $S = N - I$ with population size $N$, infection rate $\beta$. The master equation reads for the probability $p(I,t)$

$$\frac{d}{dt}p(I,t) = \frac{\beta}{N}I^* \cdot (N-(I-1))p(I-1,t) - \frac{\beta}{N}I^* \cdot (N-I)p(I,t) \tag{1}$$

which can besolved using the characteristic function

$$\langle e^{i\kappa I}\rangle := \sum_{I=0}^{N} e^{i\kappa I} \cdot p(I,t) =: g(\kappa,t) \quad . \tag{2}$$

or approximated by Kramers-Moyal expansion to preserve eventual attarctor switching in more complicated models, relevant for e.g. influenza [11].

## Solving the master equation

For suitable initial conditions the master equation can be solved to obtain the transition probabilites needed to construct the likelihood function. The dynamics of the characteristic function, obtained from the master equation is

$$\frac{\partial}{\partial t}g(\kappa,t) = \sum_{I=0}^{N} e^{i\kappa I} \cdot \frac{d}{dt}p(I,t) \tag{3}$$

giving the partial differential equation

$$\frac{\partial}{\partial t}g(\kappa,t) = \beta^* N\left((e^{i\kappa}-1)\right) \cdot g(\kappa,t) + i\beta^*(e^{i\kappa}-1) \cdot \frac{\partial g}{\partial \kappa} \tag{4}$$

using $\beta^* := (\beta/N)I^*$, and initial condition $p(I,t_0) = \delta_{I,I_0}$, hence $g(\kappa,t_0) = e^{i\kappa I_0}$, with solution

$$g(\kappa,t) = e^{i\kappa N}\left(e^{-i\kappa}e^{-\beta^*(t-t_0)} + (1-e^{-\beta^*(t-t_0)})\right)^{N-I_0} \tag{5}$$

see Fig. 1 a). Then Fourier back transformation gives the transition probability $p(I,t|I_0,t_0)$ explicitly as solution of the master equation.

## Likelihood function from master equation

>From the transitions probabilities we can construct the likelihood function, i.e. the joint probability to find all data point from our empirical time series interpreted as a function of the model parameters

$$p(I_n,t_n,I_{n-1},t_{n-1},...,I_0,t_0) = \prod_{\nu=0}^{n-1} p(I_{\nu+1},t_{\nu+1}|I_\nu,t_\nu) \cdot p(I_0,t_0) =: L(\beta) \tag{6}$$

as shown in Fig. 1 b) for the likelihood function and its maximum as best estimator $\hat{\beta}$ for the parameter $\beta$. Here the best estimator can be calculated analytically as well [3].

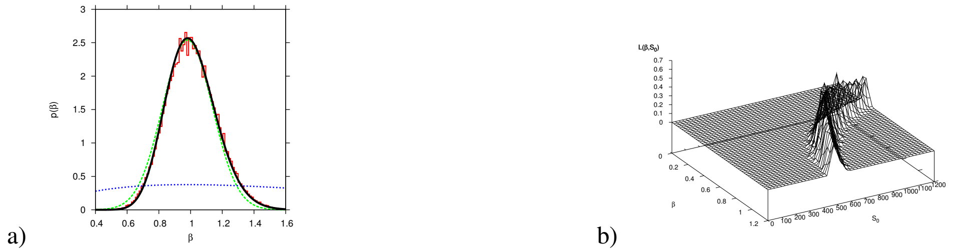

**FIGURE 2.** a) Comparison between maximum likelihood method and Bayesia approach for a simple linear infection model, with all analytical tools available. b) Application of numerical likelihood estimation of infection rate and initial number of susceptibles on influenza data from InfluenzaNet, an internet based surveillance system (EPIWORK project).

### Bayesian framework

The Bayesian framework starts by using the likelihood function $L(\beta) = p(\underline{I}|\beta)$ with $\underline{I} = (I_0, I_1, ...I_n)$ and constructs from it the probability of the parameters conditioned on the present data, here from the time series of the epidemic system, the Bayesian posterior $p(\beta|\underline{I})$. This can be only achieved by imposing a prior $p(\beta)$, a probability of plausible parameter sets, hence we have

$$p(\beta|\underline{I}) = \frac{p(\underline{I}|\beta)}{p(\underline{I})}\, p(\beta) \quad . \tag{7}$$

For the linear infcetion model all steps can still be performed analytically. The posterior is given explicitly by

$$p(\beta|\underline{I}) = \frac{\Gamma(\tilde{a}+\tilde{b})}{\Gamma(\tilde{a})\,\Gamma(\tilde{b})} \cdot e^{-\beta\Delta t} \cdot \Delta t \cdot \left(1 - e^{-\beta\Delta t}\right)^{\tilde{a}-1} \left(e^{-\beta\Delta t}\right)^{\tilde{b}-1} \tag{8}$$

with hyperparameters $\tilde{a} = a + \sum_{\nu=0}^{n-1}(I_{\nu+1} - I_\nu)$ and $\tilde{b} = b + \sum_{\nu=0}^{n-1}(N - I_{\nu+1})$ from the prior parameters $a$ and $b$. Fig. 2 a) shows the comparison of a histogram of best estimates from many realizations of the stochastic process (red), an information which is not given sufficiently in most empirical systems, often we only have a single realization, and the results from the parameter estimation, a Gaussian approximation from the best estimate and the Fisher information (green), and finally the Bayesian posterior (black) obtained from a conjugate prior (blue), which is here nicely broad, not imposing much restriction to the considered parameter values.

## NUMERICAL LIKELIHOOD VIA STOCHASTIC SIMULATIONS

In cases in which not all steps or even no step can be performed analytically, a comparison of stochastic simulations, depending on the model parameters, with the empirical data is the only available information on the parameters. In Fig. 2 b) an SIR model is fitted to empirical data from influenza, here due to the relative simplicity of the stochastic model, still a numerical enumeration of the whole relevant parameter space is computationally possible (equivalent to a flat but cut-off prior. In more complicated models, e.g. dengue fever models and data from e.g. Thailand, even a complete enumeration of the parameter space is not computationally possible, though only six parameters and nine initial conditions have to be estimated. Particle filtering, i.e. an often quite restricted distribution of parameters and initial conditions, a hard prior in Bayesian language, is stochastically integrated and compared to the empirical time series, selecting the best performers as maximum of the likelihood function.

### Application to more complex models

In such cases, in which the model can display deterministic chaos, like the one in dengue we investigate [5, 7], the short term predictability and long term unpredictability (as measured by the largest Lyapunov exponent) even prohibits the comparison of stochastic simulations with the entire time series. Hence iteratively short parts of the time series are compared with the stochastic particles, and via simulated annealing the variability of the particles is cooled down,

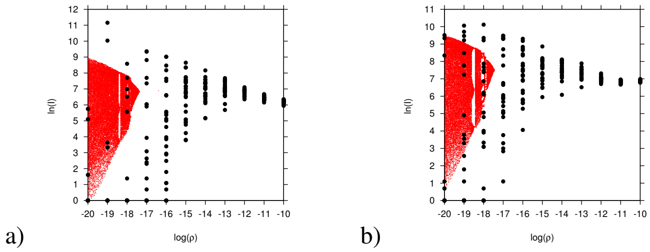

**FIGURE 3.** Comparison of deteministic skeleton and stochastic simulations for bifurcation diagrams with estimated parameters for a) dengue in Chiang Mai province and b) dengue in the surrounding nine northern provinces of Thailand. The crucial parameter is the import $\rho$. Even in systems with population sizes well above one million the noise level of the stochastic system is enormous.

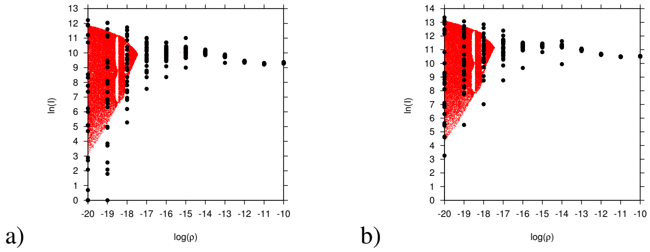

**FIGURE 4.** Extrapolation of population sizes to a) whole Thailand and b) Thailand and surrounding countries with ca. 200 mio. inhabitants. The noise level of the stochastic simulation approaches the deterministic system only for very large system sizes.

priors narrowed in Bayesian language. The final method is called "maximum likelihood iterated filtering" (MIF). We apply this method to dengue data from Thailand, and display here bifurcation diagrams obtained from the best estimates, see Fig. 3, extrapolating finally to larger population sizes, see Fig. 4.

## ACKNOWLEDGMENTS

This work has been supported by the European Union under FP7 in the EPIWORK project and by FCT, Portugal, in various ways, especially PTDC/MAT/115168/2009.
